# Incarcerated Spigelian Hernias: A Rare Cause of a High-grade Small Bowel Obstruction

**DOI:** 10.7759/cureus.7397

**Published:** 2020-03-24

**Authors:** Arye Lavin, Anupam Gupta, Miguel Lopez-Viego, Jessica L Buicko

**Affiliations:** 1 Internal Medicine, Charles E. Schmidt College of Medicine, Florida Atlantic University, Boca Raton, USA; 2 General Surgery, Charles E. Schmidt College of Medicine, Florida Atlantic University, Boca Raton, USA; 3 General and Vascular Surgery, Bethesda Hospital East, Boynton Beach, USA

**Keywords:** hernia, spigelian, small bowel obstruction, incarceration

## Abstract

A Spigelian hernia is a very rare hernia, making up approximately 0.1% of all abdominal wall hernias. This hernia goes through a defect in the Spigelian fascia which is the part of the transversus abdominis aponeurosis lateral to the rectus muscle, often at the level of the arcuate line, where the fascia is widest and weakest. We present the case of a 77-year-old female with no past surgical history who presented to our teaching hospital with high-grade small bowel obstruction secondary to an incarcerated Spigelian hernia. She was taken to the operating room for a laparotomy and a portion of the small bowel mesentery was found to be strangulated. The hernia was reduced, and the defect was repaired primarily. The diagnosis of a Spigelian hernia can often be difficult to diagnose on history and physical examination alone, but computed tomography (CT) imaging can be a valuable adjunct in diagnosis. Prompt surgical treatment should ensue when the diagnosis of high-grade bowel obstruction is made in a patient with imaging findings consistent with a Spigelian hernia.

## Introduction

The Spigelian fascia is the part of the transversus abdominis aponeurosis lateral to the rectus muscle, often at the level of the arcuate line, where the fascia is widest and weakest [1]. Hernias through a defect in this fascia account for 0.1% of all abdominal wall hernias [2]. Most patients with a Spigelian hernia are asymptomatic. If they do become symptomatic, they present with vague abdominal discomfort [3]. It is rare for patients to present with obstructive symptoms. In two large series studies (n < 100), the incidence of incarcerated Spigelian hernia was 17% and 33%, respectively [4]. Most reported incidences were only from small case reports.

We present a rare case of an incarcerated Spigelian hernia causing high-grade bowel obstruction in a 77-year-old patient presenting with nausea, vomiting, and abdominal pain.

## Case presentation

**Figure 1:**
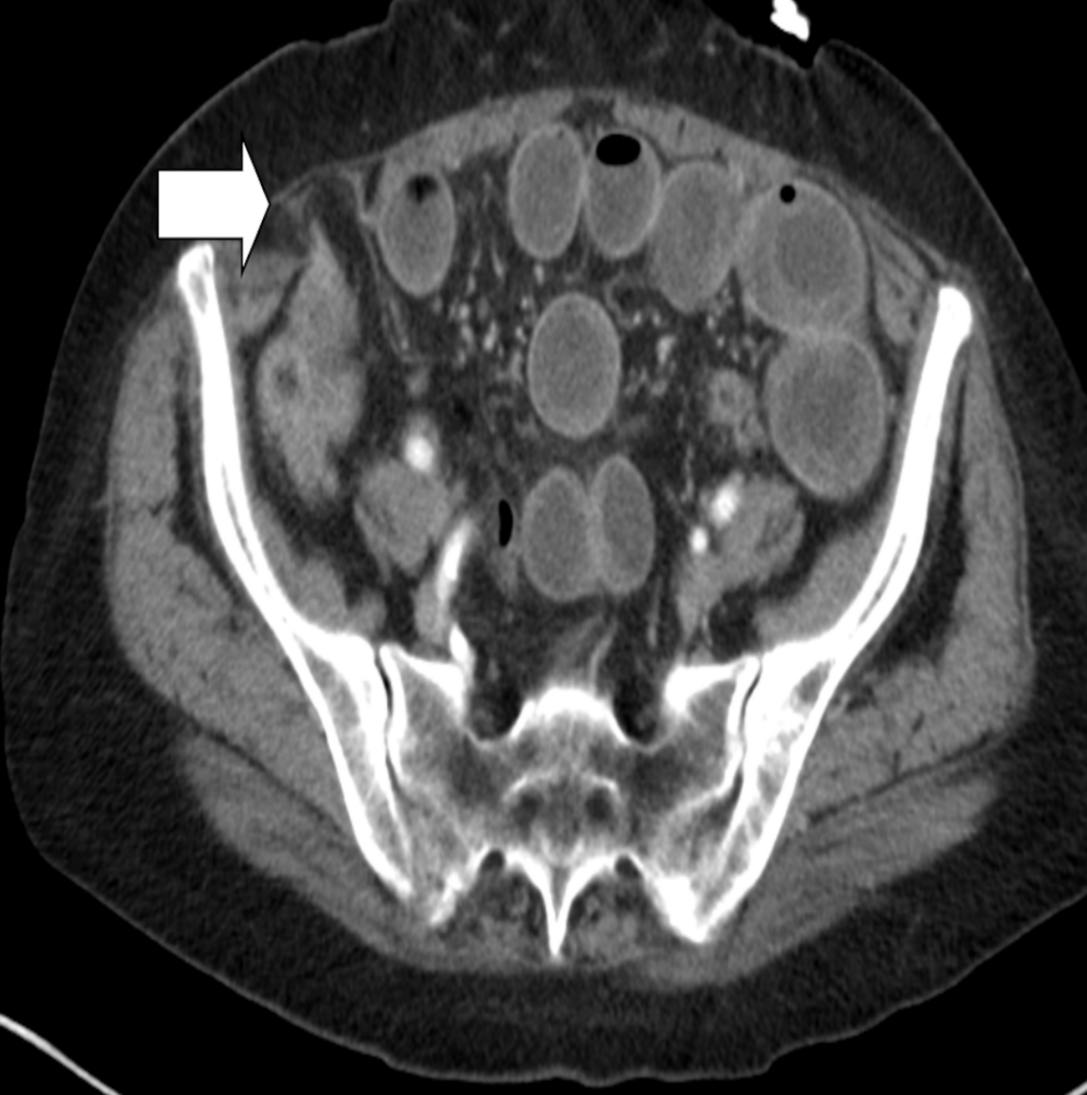
Computed tomography (CT) scan showing a Spigelian hernia defect as the transition point for small bowel obstruction (white arrow)

**Figure 2:**
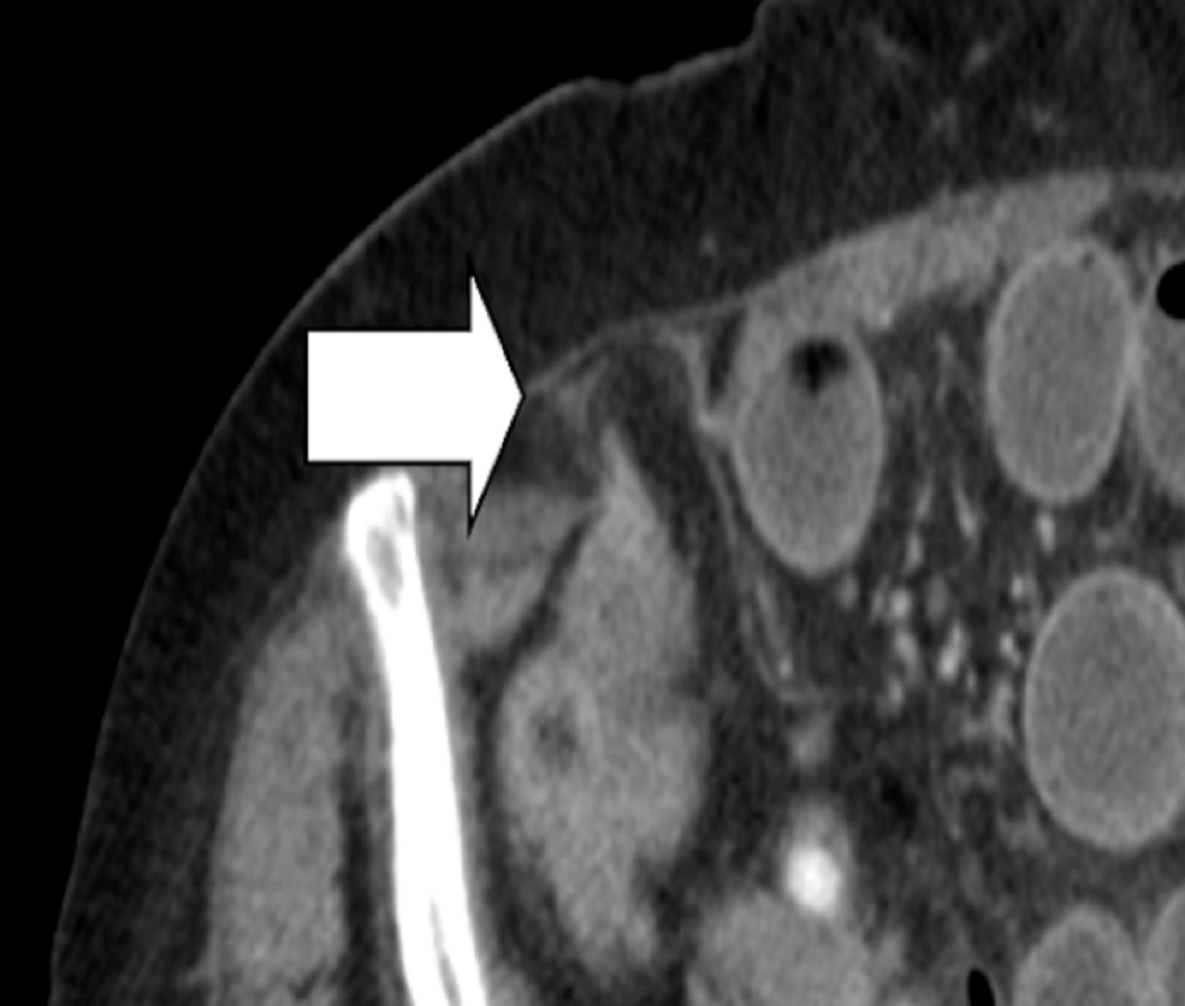
Zoom-in of the computed tomography (CT) scan showing a Spigelian hernia defect

A 77-year-old female with a past medical history of type one diabetes mellitus and hypertension presented to our teaching institution with two days of nausea, vomiting, and obstipation. She reported diffuse abdominal pain and bloating. She had no history of prior abdominal surgeries. She was hemodynamically stable on presentation. Her abdominal exam revealed a distended abdomen with generalized tenderness to palpation without rebound or guarding. She had no palpable hernias. Her laboratory work was largely unremarkable. She had a computed tomography (CT) scan performed which showed a high-grade bowel obstruction with a transition point at a right-sided Spigelian hernia (Figures 1-2). She was taken emergently for an exploratory laparotomy. Intraoperatively, a small portion of the distal small-bowel mesentery was incarcerated in the hernia and was reduced manually. The hernia defect was approximately 2 cm in size. The incarcerated area of the mesentery was bruised but the segment of the small intestine was viable. The hernia defect was repaired primarily with interrupted 2-0 polydioxanone sutures. Postoperatively, the patient’s course was uneventful as she was tolerating a regular diet by postoperative day 3 and able to be discharged home.

## Discussion

A Spigelian hernia, which was first described by Klinkosch in 1764, accounts for about 0.1% of all abdominal hernias [5]. This is a defect that occurs in the Spigelian facia located between the transversus abdominis aponeurosis lateral to the rectus muscle at the level of the arcuate line [1]. This type of hernia is most commonly seen in females with a mean age of over 60 years old [2]. It is hypothesized that perforating vessels may weaken the area in the fascia as a small lipoma or fat enters which eventually leads to hernia formation. They can also be related to stretching in the abdominal wall caused by obesity, multiple pregnancies, or previous surgeries. The hernial sac is typically intraparietal, passing through the transversus and internal oblique aponeuroses and extending out beneath the external oblique or within the rectus sheath [1]. The reported literature shows that hernia usually consists of preperitoneal fat [6]. However, the hernia may contain small bowel, colon, omentum, or occasionally, the appendix [7-8]. To our knowledge, this is the first reported case of the small bowel mesentery being incarcerated in the Spigelian hernia defect, leading to an acute small-bowel obstruction as happened in this patient. This diagnosis is usually difficult to diagnose clinically as they do not often present as a mass or bulge. Most patients present with pain as the symptom and computed tomography can be used to diagnose the hernia [9]. Most authors suggest repairing the Spigelian hernia as the fascia here typically forms a rigid neck which could lead to future incarceration or strangulation. The reported incidence of incarceration is between 24% - 27% and strangulation at 2% - 14% [6]. 

## Conclusions

A complicated obstructed spigelian hernia is an uncommon diagnosis. It is unusual to find mesentery of small-bowel being incarcerated in this Spigelian hernia defect leading to acute small-bowel obstruction. In an emergency setting, reduction of the hernia is usually sufficient if the bowel loops appear viable post reduction and CT scan confirms the diagnosis.
